# PVS-GEN: Systematic Approach for Universal Synthetic Data Generation Involving Parameterization, Verification, and Segmentation

**DOI:** 10.3390/s24010266

**Published:** 2024-01-02

**Authors:** Kyung-Min Kim, Jong Wook Kwak

**Affiliations:** Department of Computer Engineering, Yeungnam University, Gyeongsan 38541, Republic of Korea; bl43a@ynu.ac.kr

**Keywords:** time-series sensor data, synthetic data generation, time-series synthesis, IoT data generation, possibility of reproducibility

## Abstract

Synthetic data generation addresses the challenges of obtaining extensive empirical datasets, offering benefits such as cost-effectiveness, time efficiency, and robust model development. Nonetheless, synthetic data-generation methodologies still encounter significant difficulties, including a lack of standardized metrics for modeling different data types and comparing generated results. This study introduces PVS-GEN, an automated, general-purpose process for synthetic data generation and verification. The PVS-GEN method parameterizes time-series data with minimal human intervention and verifies model construction using a specific metric derived from extracted parameters. For complex data, the process iteratively segments the empirical dataset until an extracted parameter can reproduce synthetic data that reflects the empirical characteristics, irrespective of the sensor data type. Moreover, we introduce the PoR metric to quantify the quality of the generated data by evaluating its time-series characteristics. Consequently, the proposed method can automatically generate diverse time-series data that covers a wide range of sensor types. We compared PVS-GEN with existing synthetic data-generation methodologies, and PVS-GEN demonstrated a superior performance. It generated data with a similarity of up to 37.1% across multiple data types and by 19.6% on average using the proposed metric, irrespective of the data type.

## 1. Introduction

Recently, synthetic data generation has increasingly attracted attention because of its potential to significantly decrease both the cost and time associated with acquiring and labeling large amounts of empirical data. A key advantage of generating synthetic data is the ability to create diverse datasets that can be customized to specific use cases, providing a more representative sample of a target population. Moreover, synthetic data can be employed to create complex and rare scenarios that are challenging to observe in real-world data, thereby enhancing model robustness and generalization capabilities [1,2]. Additionally, synthetic data can mitigate data privacy and security concerns, as it facilitates the generation of artificial datasets devoid of sensitive information such as identifiable information, health records, and financial details. Various artificial intelligence and machine learning have observed these benefits [3,4].

**Figure 1:**
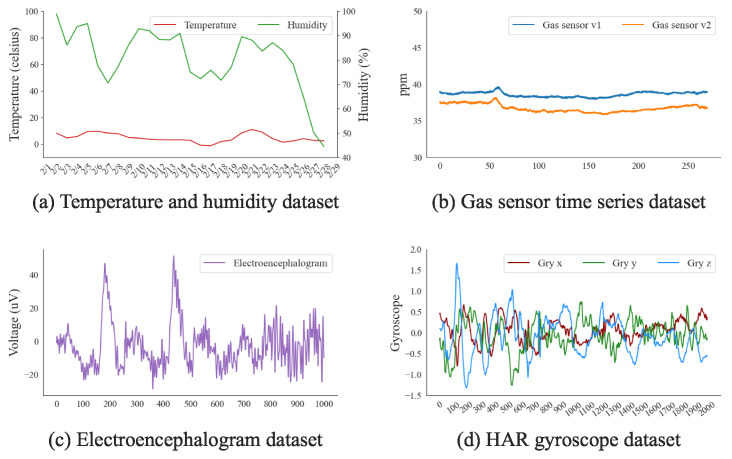
Time-series data visualizations for diverse synthetic data-generation applications across different research fields.

Figure 1 shows the various data types explored for time-series synthetic data generation. Data types (a) and (b), characterized by long measurement intervals and minimal variation, such as temperature and humidity sensor data, can be used to generate synthetic counterparts that closely mirror the empirical data; therefore, these types of data are commonly used in synthetic data generation and prediction. Data types (c) and (d) from devices such as electroencephalography (EEG) and gyroscopes, which feature short measurement intervals, significant variation, and irregular time-series, pose a considerable difficulty for modeling, particularly with the recent increase in human activity recognition (HAR) data.

The two main synthetic data-generation methods are statistical methodologies and machine learning techniques. Although relatively less expensive and precise, statistical methodologies can be restrictive in data handling and require individualized mathematical modeling processes. Moreover, their reliance on data distribution assumptions can hinder the capturing of the full complexity of real-world data [5,6,7]. In contrast, machine learning techniques, introduced in the 2010s, offer more flexibility and can handle complex data distributions. However, the efficiency and precision of these techniques can significantly depend on the learning algorithm, complicating the selection of the most suitable approach for specific applications [8,9,10].

Although various studies have been conducted on generating synthetic time-series data, they still confront numerous challenges. For example, the fragmented and ongoing nature of research in learning various time-series data remains a problem. This fragmentation complicates the generation of universal time-series data and impedes the automation of the process. Another challenge is the difficulty in accurately modeling the complexity and variability of real-world data, which can result in biases and errors in the models derived from this data. Finally, the absence of standard methods for evaluating the quality and effectiveness of synthetic data benchmarks is another problem [11,12]. The lack of universal evaluation indicators hinders the consistency of interpreting performance and comparison results. Therefore, addressing these challenges requires the development of innovative modeling methodologies, enhancement of transparency in synthetic data-generation processes, and proposal of standardized metrics for the verification and evaluation of synthetic data.

This study proposes an approach specifically designed to enhance the quality and reliability of synthetic data by incorporating three key processes: parameterization, verification, and segmentation. The parameterization process begins by defining the base data record, which is used in the synthetic data-generation and subsequent processes. This involves configuring the base data record and extracting autoregressive parameters for data generation. Next, the verification process is conducted to ensure that the synthetic data retains the properties of the empirical data. This includes verifying the similarity between the generated and empirical models, using the derived indicators as criteria for parameterization and data segmentation. Finally, the segmentation process involves subdividing the data until consistency and regularity are achieved within the partial time-series data, thereby creating a more accurate model for each segment.

In this manner, the proposed technique can generate automated synthetic data irrespective of the input data type. Furthermore, this study proposes an evaluation methodology intended for standardized comparisons of synthetic data-generation techniques. This approach addresses issues related to performance factors and insufficient comparison standards, thereby offering a more transparent evaluation of synthetic data benchmarks.

The remainder of this paper is organized as follows. The following Section 2 reviews previous studies on synthetic data-generation methodologies and outlines our research objectives. We introduce the proposed methodology for synthetic data benchmark generation and examine the features of each process in Section 3. Section 4 analyzes the results obtained from the proposed synthetic data benchmark generation system. Finally, Section 5 concludes the paper.

## 2. Background and Related Works

### 2.1. Overview of Synthetic Time-Series Data Generation

Recently, research on the creation of synthetic data benchmarks across various fields, including sensor technology, health informatics, and financial modeling has been increasingly in demand. Three main factors drive this demand [13,14,15,16].

First, the volume of raw data being acquired has been steadily increasing because of the rise of the Internet of Things (IoT) and advancements in sensor informatics technology. Consequently, the number of fields requiring various types of data processing has surged. However, the measured data used in current studies may be unrefined or missing, making the obtainment of expected data for the targeted environment challenging. Appropriately generated synthetic data can help address these issues and assist in performance evaluations and data processing characterizations.

Second, synthetic data can be utilized to train machine learning models and build decision-making models that require large amounts of training data. The use of multiple open data platforms, such as Kaggle, Google Dataset Search, and UCI Machine Learning Repository, often results in subpar model performances owing to the lack of dataset diversity and high heterogeneity. To generate high-quality synthetic data, companies such as NVIDIA and IBM, as well as government agencies, are adopting various data synthesis methodologies [17,18,19].

Third, an escalating demand for non-identifying data exists. With the general-purpose use of data, sensitive personal information can be included in collected data, which raises privacy concerns. The need for de-identification has emerged, and identifying individuals through data combinations is difficult. In the medical and healthcare sectors, data analysis often requires the highest level of granularity. Stringent data collection regulations can present significant hurdles in this regard. Consequently, solutions that can navigate between the dual imperatives of in-depth data analysis and robust privacy protection are in urgent need. Synthetic data have emerged as an alternative that can provide effective data while satisfying the de-identification condition, as the data are statistically similar to the empirical data [13,14,16,20,21,22,23,24,25].

Nonetheless, challenges remain in time-series synthetic data research, which include the difficulty in modeling some time-series data owing to data-specific characteristics. For instance, statistical modeling involves data types that make creating a model for generating synthetic data difficult, particularly for data types with significant variances in sensor data [11,26]. To address this, we classified these data types according to their frequency, variability, and regularity, as shown in Table 1.

- Frequency denotes the number of data inputs collected during a specific time frame. High-frequency data, characterized by more values per unit of time, typically display increased variability and regularity. Conversely, low-frequency data exhibit decreases in these traits.
- Variability measures the degree of change in sensor values over time. A high variability indicates rapid and frequent changes in the sensor values, whereas a low variability denotes slow and gradual shifts.
- Regularity refers to the uniformity of the recurring patterns in the data. High regularity suggests a consistent pattern, requiring fewer data samples to fully represent the data. Conversely, low regularity indicates less uniformity in the data patterns, requiring more data samples for a complete representation.

**Figure 2:**
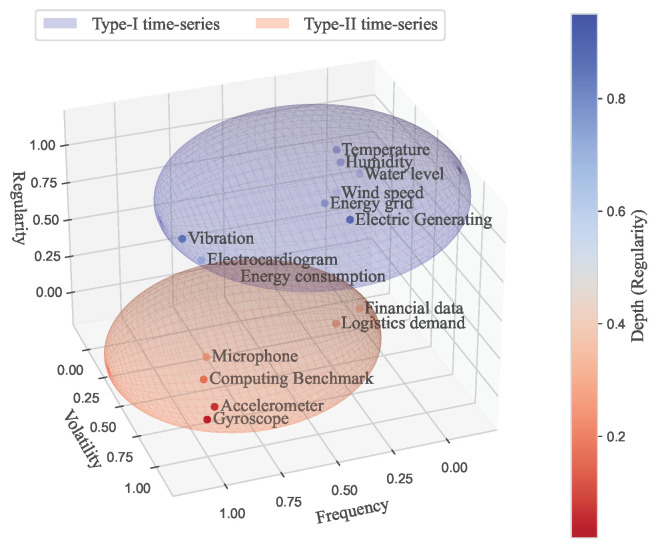
Categorization of time-series data into Type-I and Type-II for research topics.

Figure 2 shows the classification of data types that are the subject of synthetic data generation, based on Table 1. Numerous previous studies have generated synthetic data corresponding to Type-I. However, synthetic data types associated with Type-II data receive less attention; most of the existing discourse has focused on forecasting Type-I time-series data. Synthesizing Type-II presents difficulties when utilizing a single model. However, the demand for this data type is steadily increasing owing to advancements in sensor technology and the rapid proliferation of IoT-based devices. This study aims to identify conditions for universal data-generation techniques that can be applied to both Type-I and Type-II data, particularly focusing on data with complex time-series characteristics.

### 2.2. Related Works

Time-series synthetic data are generated for several purposes, including (1) sensor performance evaluation, (2) dataset amplification, or (3) data de-identification. To address these purposes, various techniques for synthetic data generation have been proposed. These techniques can be classified into two categories as follows.

- Statistical methods: Traditional statistical methods have been employed to generate time-series synthetic data by modeling sensor output results. Examples of statistical models include simple exponential smoothing (SES), autoregressive integrated moving average (ARIMA), and Gaussian mixture models (GMM).

Statistical methods offer several benefits, such as allowing the definition of suitable parameters to facilitate efficient synthetic data generation. They also negate the need for separate learning or data processing, resulting in less overhead operations in the data analyses and measurements. Furthermore, statistically modeled data can be used for various tasks, such as in processing, forecasting, and verification, using pre-established mathematical models.

However, these methods have some limitations. They can seem rigid and inflexible owing to their dependency on specific assumptions regarding the data distribution, which may render them less adaptable and require unique models for different synthetic data types when these assumptions are invalid. Additionally, estimating models for complex time-series data can require substantial qualitative effort, posing challenges in effective automation. Nonetheless, statistical methods continue to play an essential role in generating time-series synthetic data, particularly when the data exhibit clear statistical patterns and a high stationary degree [27,28,29,30,31,32,33,34,35,36,37].

- Machine learning methods: These techniques leverage machine learning algorithms to learn patterns and structures in the empirical data and generate synthetic data that resemble the original data. Examples of machine learning methods for synthetic data generation include deep learning techniques such as generative adversarial networks (GANs), variational autoencoders (VAEs), support vector regression (SVR), and recurrent neural networks (RNNs).

Machine learning methodologies offer advantages such as the ability to flexibly handle complex data distributions, which is superior to statistical methods. In addition, they minimize non-quantitative tasks such as parameter selection and tuning, allowing for in-system optimization and parameter calculation. Moreover, these methods are robust against outliers and permit flexible data adjustments owing to their reduced sensitivity to input data shapes.

However, machine learning methods also have limitations. They suffer from learning rate issues such as overfitting, particularly when handling sensor data with significant fluctuations, making them less ideal for data with unusual variance. Data model sharing and usage can be restricted in environments processing various sensor data types owing to potential increases in the model size and associated data. Additionally, establishing consistent performance indicators may be challenging as the final output depends more on a specific learning model or methodology than a standard mathematical model. While machine learning methods have certain constraints, they have demonstrated significant potential in generating realistic and diverse synthetic data and applicability across a broad range of uses. The recent studies on statistical and machine learning methods are summarized in Table 2 [38,39,40,41,42,43,44,45,46,47].

### 2.3. Challenges and Contributions

Many existing studies on synthetic data generation have failed to establish a model that can universally accommodate various types of time-series data. Although such studies are conducted individually across diverse data types, model generalization and process automation challenges remain owing to the need to conduct supervised learning or model tuning depending on the data type. Consequently, the research becomes fragmented, and comparing performances across different methods is difficult. Current methods tend to visually compare empirical and synthetic data distributions, which is an approach that can inadequately capture the full characteristics of diverse data types. Other attempts have been made to use descriptive statistics, such as the mean and root-mean-square error, for data comparisons. However, these methodologies remain sensitive to the number of generated data points and provide improper error representations [11,48,49].

To address these issues, our research proposes the following:

- A universal synthetic data-generation model independent of sensor data traits.
- An automated process for data generation that eliminates the need for parameter estimation or separate supervised learning.
- A universally applicable verification metric irrespective of sensors and generation methodologies.

By proposing a synthetic data-generation model and automated process that are independent of sensor data characteristics, we address the current limitations and provide a more efficient approach. Moreover, the introduction of a universally applicable verification metric enhances the consistency and reliability of future data evaluation.

Our contributions are summarized as follows:

- We introduce a modeling methodology for universal synthetic data generation that is independent of sensor data traits, thereby enabling adaptable data synthesis for diverse sensor types and a generation process with unparalleled scalability.
- We propose an automated process with its data frame for synthetic data generation, thereby reducing the intricacies of parameter tuning and supervised learning, which bolsters consistency, enhances reproducibility, and streamlines the qualitative overhead in the modeling process.
- We formulate a universally applicable verification metric that adeptly encapsulates the temporal dynamics of time-series data, facilitating precise differentiations between empirical and synthetic datasets, thereby augmenting both the consistency and reliability of data quality assessments across various research endeavors.

## 3. PVS-GEN: Automated Universial Synthetic Data Generation

**Figure 3:**
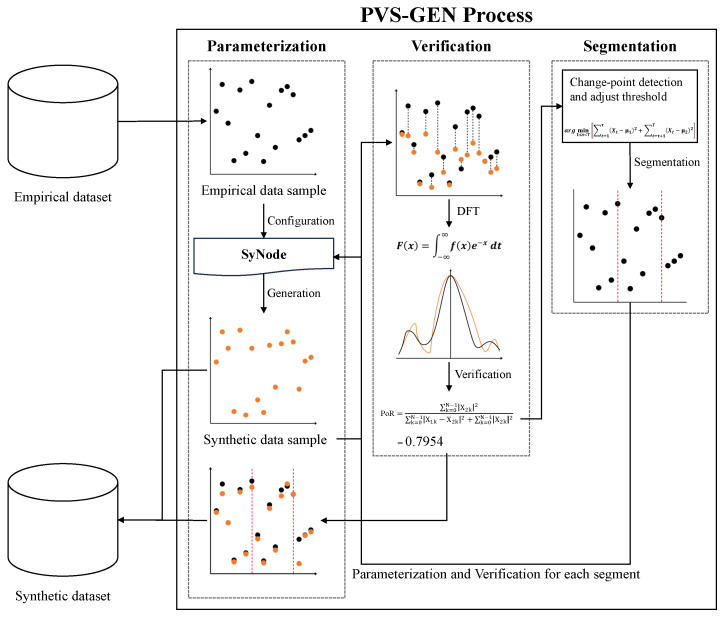
Overview of the PVS-GEN process.

This section introduces the proposed PVS-GEN process: parameterization, verification and segmentation for universal synthetic data generation. This automated method generates synthetic data from general-purpose time-series data of any type. Figure 3 overviews the steps of the PVS-GEN process, demonstrating the transformation of empirical data into synthetic benchmarks.

The PVS-GEN begins with parameterization process. In this step, we utilize empirical data with ARIMA to derive automated parameters, effectively reducing user intervention. Subsequently, the generated data undergo a verification process. During this verification, we compare the synthetic data with the empirical data using our proposed metric, the possibility of reproducibility (PoR). If the model fails to satisfy the PoR criterion, we employ segmentation to enhance the time-series consistency and regularity, thereby augmenting its suitability for ARIMA modeling. This three-step process is recursively performed to derive the optimal parameters to generate synthetic data. The following subsections further detail this process.

### 3.1. Parameterization: Configuring a SyNode for Synthetic Data Generation

**Figure 4:**
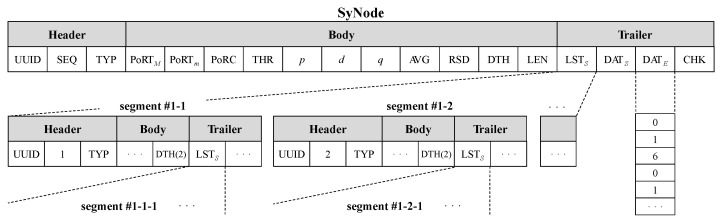
Data frame structure of a SyNode.

In the parameterization process, we construct the fundamental data frame necessary for the entire PVS-GEN process and extract ARIMA parameters from the given empirical data to generate synthetic data. In the first step, we construct the data frame, which we call a synthetic node (SyNode) in this paper. SyNodes serve as foundational data frames optimized for generating the synthetic time-series data. A SyNode integrates identifiers for the time series, parameters for synthesis, statistical metrics for verification, and the empirical source data. The SyNode is used in the parameterization process and can be used in the subsequent verification and segmentation processes, where its data values can be updated. Figure 4 shows the key elements of a SyNode.

Figure 4 shows the overall structure of a SyNode. The SyNode comprises three main sections: Header, Body, and Trailer. The Header encompasses unique identifiers: SEQ for maintaining time-series data sequences and TYP for various sensor types; for example, 1 signifies temperature, 2 signifies gas, and 3 signifies acceleration sensor data.

The Body section of a SyNode encompasses various parameters derived from the ARIMA process, along with several statistical information extracted from the empirical data embedded within the SyNode. Notably, parameters such as ${\mathrm{PoRT}}_{M}$, ${\mathrm{PoRT}}_{m}$, PoRC, ARIMA parameters *(p, d, q)*, AVG, RSD, DTH, and LEN are prominently utilized in the PVS-GEN process. ${\mathrm{PoRT}}_{M}$ and ${\mathrm{PoRT}}_{m}$ define the upper and lower bounds of the target to be replicated by the model generated using the extracted ARIMA parameters. Typically, the model aims for ${\mathrm{PoRT}}_{M}$. However, it can flexibly reduce the model generation cost based on the ratio of the threshold (THR). PoRC, produced during the verification process, is utilized to confirm the reproducibility of the SyNode parameters by examining whether its value resides within the ${\mathrm{PoRT}}_{m}$ and ${\mathrm{PoRT}}_{M}$ bounds. The *p*, *d*, and *q*, extracted from the parameterization process, are utilized in the ARIMA model to generate synthetic data. RSD denotes the residuals possessed by the empirical data and is used to reflect the noise of the residuals during the ARIMA process. In addition, it records the data length (LEN) of the current node and depth (DTH) of the node owing to recursive construction. Moreover, the Body section includes basic descriptive statistics such as the maximum, minimum, and average.

The Trailer section stores raw data and ensures data integrity. During the segmentation process, the divided sub-nodes are denoted as a self-referential structure, ${\mathrm{LST}}_{S}$, and used as the entry point for subsequent modeling processes. Furthermore, the Trailer section includes synthetic data (${\mathrm{DAT}}_{S}$) created by the ARIMA process and empirical data (${\mathrm{DAT}}_{E}$) for the extraction parameters. It contains the raw values of each synthetic and empirical data point. To verify the integrity and authenticity of the SyNode, CHK contains a unique value calculated using MD5 checksum.

This study utilized the Hyndman–Khandakar algorithm for automated ARIMA parameterization to optimize temporal data forecasting. The ARIMA model was constituted using three parameters, *p*, *d*, and *q*, as derived from Equation  (1):

(1) $$\left(1-\sum_{i=1}^{p}\phi_{i}L^{i}\right){\left(1-L\right)}^{d}Y_{t}=\left(1+\sum_{i=1}^{q}\theta_{i}L^{i}\right)\epsilon_{t}$$

where $Y_{t}$ is a data point at time *t*. The model achieves stationarity by differencing (*d*). Parameters *p* and *q* denote the influence of past observations and errors, respectively. Model selection utilizes the Akaike information criterion (AIC) or Bayes information criterion (BIC), making the ARIMA calibration less intricate [50]. To synthesize the time-series data, we constructed an ARIMA model using SyNode data. The model employs parameters *p*, *d*, *q*, and RSD, representing the autoregressive component, differencing degree, moving average component, and residuals, respectively. This is shown in Equation (2):

(2) $$X_{t}=c+\sum_{i=1}^{p}\phi_{i}X_{t-i}-\sum_{i=1}^{d}X_{t-i}+\sum_{i=1}^{q}\theta_{i}\epsilon_{t-i}+\epsilon_{t}$$

where $X_{t}$ is the predicted series value at time *t*, computed from past data points ($X_{t-i}$), past forecast errors ($\epsilon_{t-i}$), and a constant *c*. The forecast error, $\epsilon_{t}$, represents the RES or difference between the actual and forecasted values. This model, when fitted to empirical data, enables the generation of synthetic data exhibiting similar statistical patterns [51]. Consequently, the SyNode, which comprises diverse statistical information, serves as an optimized model of the empirical data. This enables the generation of multiple similar synthetic datasets, reducing user intervention and reliance on data characteristics.

### 3.2. Verification: Assessing Model Reproducibility via Frequency Domain Analysis

The verification process aims to assess the reproducibility of the empirical data generated by the model created. It transforms both the input empirical data and generated model into frequency components and then compares them using the discrete Fourier transform (DFT). This process can be conducted to quantitatively assess the results of the generated data compared with the time-series data or all PVS-GEN steps independently. Consequently, we propose the PoR metric for measuring the similarity between two time-series datasets.

The verification process begins by interpolating the input data to ensure consistent lengths; this is a prerequisite for performing the DFT. Notably, synthetic data are often generated with a length that differs from that of the empirical data. Given these variations in data length, we applied an interpolation method, as outlined in Equation (3).

(3) $$x_{i}=x_{i-1}+\frac{\left(x_{i+1}-x_{i-1}\right)}{2}$$

Equation (3) interpolates a new data point at index *i* in a time series based on adjacent data points $x_{i-1}$ and $x_{i+1}$, calculating the midpoint between the preceding data point ($x_{i-1}$) and subsequent data point ($x_{i+1}$). We used this procedure to equate the lengths of two signals before further processing. Next, we applied the DFT to both time-series datasets, translating them from the time domain to the frequency domain. We computed the Fourier coefficients using Equation (4):

(4) $$X_{k}=\frac{1}{2N}\left|\sum_{n=0}^{N-1}x_{n}e^{-i2\pi kn/N}\right|$$

where $X_{k}$ denotes the Fourier coefficient of the $k_{th}$ frequency component, calculated by summing all data points $x_{n}$ in the time-domain signal; and *N* is the total number of data points. Each $x_{n}$ is multiplied by a complex exponential term, which is a function of data point index *n*, frequency component index *k*, and *N*. We divide the absolute value of this complex sum by 2N to normalize the magnitude. Subsequently, we compute the PoR to assess the spectral similarity between the two signals, as defined in Equation (5):

(5) $$\mathrm{PoR}=\frac{\sum_{k=0}^{N-1}{\left|X_{2k}\right|}^{2}}{\sum_{k=0}^{N-1}{\left|X_{1k}-X_{2k}\right|}^{2}+\sum_{k=0}^{N-1}{\left|X_{2k}\right|}^{2}}$$

The PoR estimates the spectral similarity between two signals by calculating the ratio of the sum of squares of the Fourier coefficients of the second signal $X_{2k}$ to the sum of square differences of the Fourier coefficients of the first and second signals $X_{1k}$ and $X_{2k}$, plus the sum of squares of the Fourier coefficients of the second signal $X_{2k}$. The PoR yields a ratio that numerically represents the similarity between the Fourier coefficients of the two signals, where a score near 1 indicates high similarity. Conversely, a score near 0 suggests that the two datasets represent an opposite time-series characteristic. Using the PoR facilitates the quantification and comparison of the similarity in time-series data by considering their temporal characteristics. Additionally, it mitigates issues that can arise from varying data counts, employing interpolation.

Algorithm 1 summarizes these steps and provides an efficient method for quantifying similarity. Algorithm 1 first equalizes the lengths of the two input datasets by interpolating the shorter dataset to match the length of the longer one (lines 1–4). Subsequently, a DFT is performed on both datasets, transforming the time-series data into the frequency domain and creating two new variables: $FFT_{D_{1}}$ and $FFT_{D_{2}}$ (line 5). Next, the norm square of the second dataset ($NormSq_{D_{2}}$) is calculated (line 6), and the sum of the squared differences between the Fourier transforms ($SumSq_{\delta }$) of both datasets is computed. These computed values determine the PoR value (lines 7–8), and the algorithm finally returns the derived PoR value (line 9).
**Algorithm 1** Obtain time-series similarity via the possibility of reproducibility
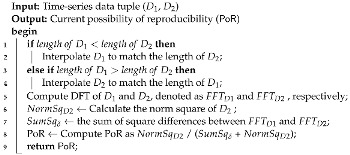


### 3.3. Segmentation: Data Partitioning with Change-Point Detection

Time-series data often represent high volatility and complex temporal changes, making the computation of a model predicated on a singular parameter set challenging. To mitigate this, we divided the given time-series data into multiple segments, allowing for the extraction of suitable parameters. The segmentation process aims to subdivide the data until time-series consistency is achieved within the partial time-series data, thereby obtaining a more accurate model of each segment via the parameterization process.

We employed a multiple change-point detection method to segment the time-series data. This statistical technique identifies points where the time-series characteristic changes significantly. For a univariate time series $X={\left(X_{t}\right)}_{t\geq 1}$, multiple change points $\tau_{1},\tau_{2},\ldots ,\tau_{k}$ exist such that the distributions of each segment $\left(X_{1},\ldots ,X_{\tau_{1}}\right),(X_{\tau_{1}+1},\ldots ,$$X_{\tau_{2}}),\ldots ,\left(X_{\tau_{k}+1},\ldots ,X_{T}\right)$ differ. We detect these change points by iteratively applying the following equation to the series and its segments:

(6) $$\tau^{*}=\arg\min_{1\leq \tau <T}\left[\sum_{t=1}^{\tau }{\left(X_{t}-\mu_{1}\right)}^{2}+\sum_{t=\tau +1}^{T}{\left(X_{t}-\mu_{2}\right)}^{2}\right]$$

where *T* is the series length, $\mu_{1}=\frac{1}{\tau }\sum_{t=1}^{\tau }X_{t}$ is the mean up to τ, and $\mu_{2}=\frac{1}{T-\tau }\sum_{t=\tau +1}^{T}$ $X_{t}$ is the mean after τ. This method allows us to efficiently identify significant shifts and segment the data for an improved parameterization process. By integrating this multiple change-point detection method in our algorithm, we can identify potential outliers and segment the data more efficiently [52,53].

The segmentation process facilitates the tuning of suitable parameters for given time-series data. However, it may impose an overhead by increasing the total number of PVS-GEN process iterations. To mitigate excessive segmentation processes across specific areas and enhance the overall efficiency of the process, we implemented the following two strategies. First, we set a maximum threshold for the number of segmentation processes that can proceed. Using a defined threshold within the SyNode, if segmentation becomes excessively deep for a specific data point, we designate that area as a fail instead of a fit. This approach ensures that room is available for further analysis and the application of more suitable model-fitting methodologies. Second, we designed the process such that the target PoR value can be adjusted flexibly within the minimum and maximum limits based on the progress of the segmentation process. The convergence of the number of segmentations to the threshold value implies that (1) the particular model struggles with time-series modeling using ARIMA and (2) the segmented subintervals are becoming shorter; thus, the accuracy of a single interval does not significantly impact the overall process. Hence, we allow the PoR threshold value to fluctuate between the upper and lower bounds (${\mathrm{PoRT}}_{M}$ and ${\mathrm{PoRT}}_{m}$, respectively) depending on the threshold (THR). We define this flexibility as η, which can be expressed using Equation (7):

(7) $$\eta ={\mathrm{PoRT}}_{M}-\Delta \mathrm{PoRT}\cdot \frac{1}{1+e^{-\frac{1}{\sqrt{\mathrm{THR}}}\times \left({\mathrm{count}}_{seg}-\mathrm{THR}/2\right)}}$$

where ΔPoRT denotes ${\mathrm{PoRT}}_{M}-{\mathrm{PoRT}}_{m}$. η gradually transitions to a model with a lower threshold as the segmentation count (${\mathrm{count}}_{seg}$) approaches the threshold value, converging from ${\mathrm{PoRT}}_{M}$ to ${\mathrm{PoRT}}_{m}$. To correspond to a nonlinear monotonically increasing function within the upper and lower bounds, ΔPoRT is defined as a function with a sigmoid-shaped slope. The THR parameter, as presented in the equation, is crucial for effectively managing the rate of change in η and ensuring a smooth and controlled transition as ${\mathrm{count}}_{seg}$ varies. Furthermore, this parameter is pivotal in regulating abrupt changes in the segmentation process by adjusting the slope of the sigmoid function. These strategies prevent excessive segmentation that can result from the segmentation process and provide a balance between the performance overhead and quality of the generated data.

Algorithm 2 shows the proposed data segmentation algorithm. This algorithm divides the empirical dataset into multiple segments, thereby maintaining time-series consistency for the parameterization process. Algorithm 2 first verifies whether the segment list (${\mathrm{LST}}_{S}$) is initialized. If it is, the ${\mathrm{LST}}_{S}$ is populated with the empirical dataset (${\mathrm{DAT}}_{E}$) to establish a basis for the segmentation process (lines 1–2). Each invocation of the segmentation process results in an adjustment of the η value, as expressed in Equation (7). η is adjusted to a value between the minimum and maximum of PoRT based on the current threshold. Hence, ${\mathrm{PoRT}}_{M}$ serves as an upper bound, which is used to calibrate the maximum value compared with PoRC (lines 3–4). The algorithm then employs a change-point detection function on the ${\mathrm{DAT}}_{E}$, yielding a collection of *n* change points (CPs), considering significant shifts in statistical data properties, which delineate distinct data segments (lines 5–6). For each iteration *i*, a new segment instance (${\mathrm{segment}}_{i}$) is formed and its parameters are updated accordingly. The start and end points of each segment are set based on *i*. In particular, if i>0, the Head is assigned the value of the CP at i−1; otherwise, it is set to 0. If i<n−1, the Tail is set to the CP at *i*; otherwise, it is set to the length of the data. ${\mathrm{segment}}_{i}$ is defined as the subset of the ${\mathrm{DAT}}_{E}$ from the Head to Tail and added to a list of new segments (lines 7–11). After generating all new segments, the algorithm discards the existing ${\mathrm{DAT}}_{E}$ from the ${\mathrm{LST}}_{S}$ (line 12). Finally, the algorithm returns the updated ${\mathrm{LST}}_{S}$ that now includes the segmented ${\mathrm{DAT}}_{E}$ (line 13).
**Algorithm 2** Divide a SyNode into SyNode segments with change-point detection
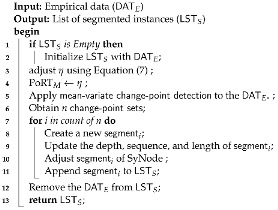


### 3.4. PVS-GEN Process

**Figure 5:**
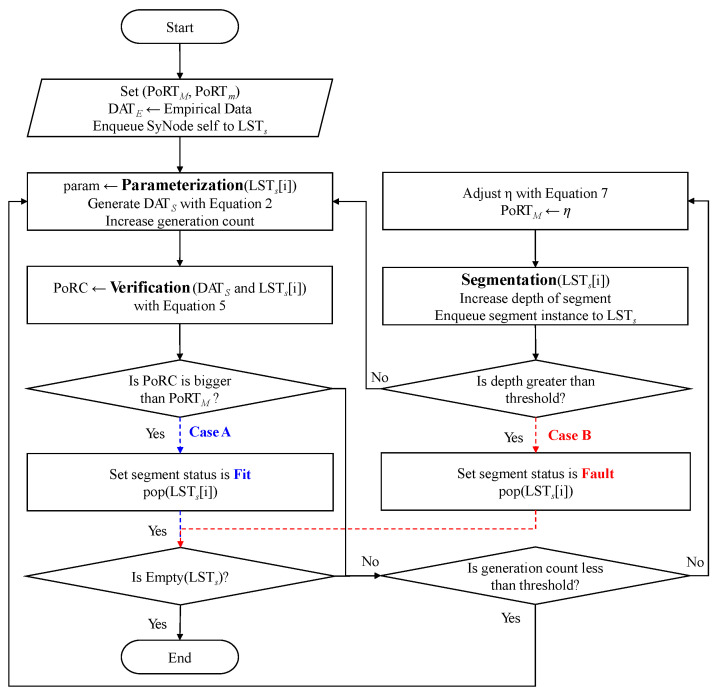
Flowchart of the overall PVS-GEN process.

To offer a comprehensive perspective on the PVS-GEN process, we present detailed flowcharts. Figure 5 shows the overall PVS-GEN process including from the parameterization process to the segmentation process. As shown in Figure 5, the PVS-GEN process first configures the SyNodes that fit the empirical data and the goals of the model. The subprocesses execute while the ${\mathrm{LST}}_{S}$ is not empty, ultimately defining the model as a fit (Case A) or fault (Case B) In the PVS-GEN process, several subprocesses execute iteratively. For each iteration, a SyNode is taken from the ${\mathrm{LST}}_{S}$, and these subprocesses execute until the ${\mathrm{LST}}_{S}$ is empty.

In each iteration, the model either fits or does not fit the data (Cases A and B, respectively). In Case A, the process continues to the next SyNode segment in the ${\mathrm{LST}}_{S}$. In Case B, the respective segment is marked as a region not used for further processing. This can occur if the current model fails to fit the segment adequately; it can be stored separately for future reference and analysis.

This iterative PVS-GEN process continues until no more segments are in the ${\mathrm{LST}}_{S}$, thereby ensuring that the synthetic data are well represented by the model. The outcome of this process is a synthetic dataset that reflects the statistical properties of the original empirical data, adhering to the objectives set at the beginning of the process.

Based on the PVS-GEN methodology proposed in our research, the principal contributions can be summarized as follows:

- Innovative methodology for synthetic data generation: PVS-GEN builds upon existing methods of synthetic data generation but refines and enhances them specifically for the challenges of time-series data generation. This approach has led to the development of a novel methodology capable of effectively generating synthetic data that mirror the empirical characteristics across various sensor data types.
- Automated synthetic data generation process: PVS-GEN introduces an automated process for data parameterization, significantly reducing human intervention. Simultaneously, it incorporates an efficient segmentation and parameter extraction process for empirical datasets. This approach allows for the effective handling of complex data and enhances the consistency and regularity of time-series data.
- Introduction of a new metric for time-series similarity evaluation: Our research proposes the PoR metric as a novel method for evaluating the quality of synthetic data. This metric assesses time-series characteristics in a manner different from existing models, offering a new standard for evaluating the quality of synthetic data. It enables researchers to compare the similarity between synthetic and empirical data more precisely.

## 4. Results and Discussion

This study procured subsets from diverse categories of empirical data and generated synthetic data. We evaluated the reproducibility of the synthetic data generation of our proposed PVS-GEN process using several time-series datasets with different time-series characteristics. We assessed the performance of our synthetic data-generation method based on the statistical significance of the time-series characteristics inherent in the target empirical data. Note that, owing to the stochastic process, the synthetic data generation yielded different data values every iteration. Finally, our evaluation involved comparing the PoR values and ensuring data distribution consistency through multiple iterations.

### 4.1. Experiment Setup

For the performance evaluation, we selected datasets from the UCI repository, which encompasses various types of data, such as multivariate, univariate, sequential, and text data, including time-series data, used for artificial intelligence and data science. The following UCI time-series datasets were used:

- Gas Sensor Array Dataset: This dataset, generated from a small polytetrafluoroethylene (PTFE) test chamber exposed to dynamic mixtures of CO and humid synthetic air, provides the mixtures sourced from high-purity gases and delivered to the chamber through a mass flow-controlled piping system [54].
- Low-Energy House Dataset: This dataset contains data on the energy usage of appliances in a low-energy house. It features both temperature and humidity measurements, providing a comprehensive account of energy consumption patterns in relation to these environmental variables [55].
- EEG Alcoholism Dataset: This dataset is from a study investigating how EEG signals correlate to the genetic predisposition of alcoholism. The dataset features EEG recordings from 122 subjects, categorized as alcoholic or control. Subjects were exposed to visual stimuli under various conditions. Three versions of the dataset are available, differing in the number of subjects and trials included [56].
- Heterogeneity Activity Recognition Dataset: This dataset includes readings from two motion sensors (Accelerometer and Gyroscope) in smartphones and smartwatches, used to investigate sensor heterogeneities’ impacts on human activity recognition algorithms. Data were collected from nine users executing six activities, capturing data at the highest frequency allowed [57].

In our experiment, each methodology was implemented in Python. The statistical models, SES and ARIMA, utilized automated parameter application from the statsforecast library to generate synthetic data. For the SVR model, the hyperparameters were set as follows: gamma, 0.1; regularization parameter C, 1e4; and the Gaussian kernel was used. The LSTM model was configured with 100 hidden layers, a batch size of 10, and was trained for 256 epochs. The proposed PVS-GEN technique was configured with a threshold of 100, ${\mathrm{PoRT}}_{m}$ of 0.85, and ${\mathrm{PoRT}}_{M}$ of 0.95. The experiments were conducted in a computing environment featuring an AMD Ryzen 9 5900X 12-Core Processor with 64 GB of RAM. To provide a clear overview of the experimental setup, we have summarized the empirical data in Table 3 [54,55,56,57,58].

### 4.2. Results and Discussion

Several studies frequently compare the descriptive statistics of synthetic data and visualize the generated data to evaluate their synthetic data-generation techniques. Comparing the actual data-generation models enables the assessment of how well the generation techniques reflect the empirical data and evaluation the validity of using synthetic data across various datasets [30,31,39,59,60,61].

**Figure 6:**
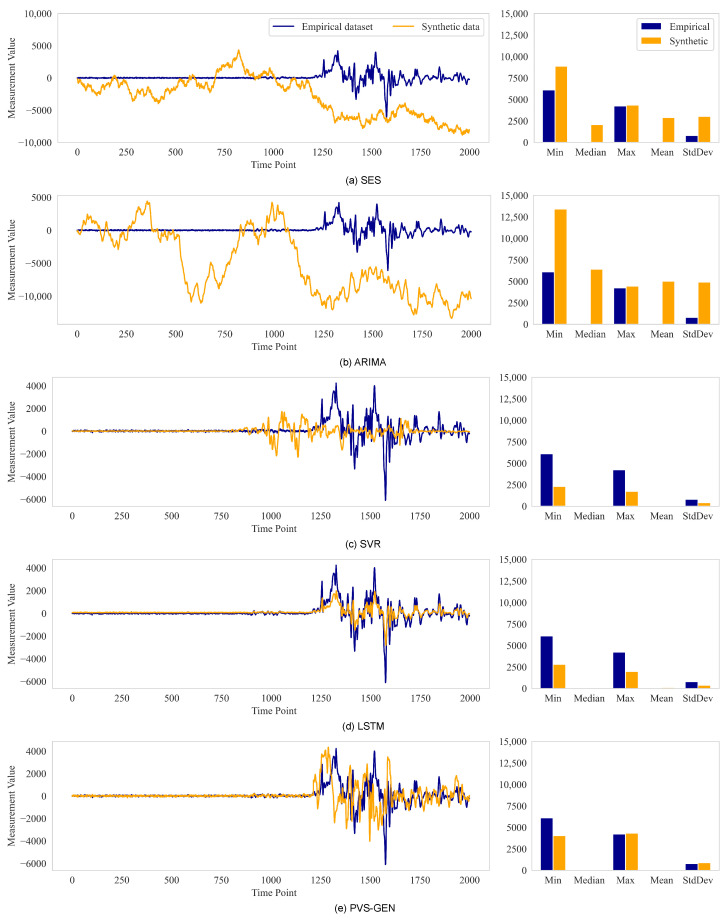
Visualizations of empirical and synthetic data with their descriptive statistics: blue lines represent generation data from SES, ARIMA, SVR, LSTM, and PVS-GEN models, while orange lines indicate empirical data.

Figure 6 shows the results of visualization and statistical analysis, which were performed on synthetic data generated from the sampled time-series data. The SES and ARIMA methods (Figure 6a,b) struggled to properly reflect the data, particularly in ranges in which values and characteristics changed rapidly. Conversely, the SVR and LSTM models (Figure 6c,d) employed machine learning methodologies to better reproduce the corresponding sections. However, the machine learning methodologies did not properly reflect rapid changes in the values. This resulted in a large discrepancy between the maximum and minimum values of the descriptive statistics. The proposed PVS-GEN method (Figure 6e) segmented the time-series data and performed ARIMA operations on each segment. This approach more accurately reflected the characteristics of the time-series data, even for rapid value changes, as shown in the descriptive statistics.

Descriptive statistics can confirm the statistical numerical distribution of the synthetic data; however they are limited in interpreting the tendency of data changes in the time domain, as discussed in Section 2. The synthetic data-generation process is a probabilistic statistical process, and the accuracy of individual factors for different data generations cannot be guaranteed by a single experimental result. Therefore, we verify how the proposed PoR metric reflects the technical statistics of the actual data. Subsequently, we use it to compare performances across various methodologies.

**Figure 7:**
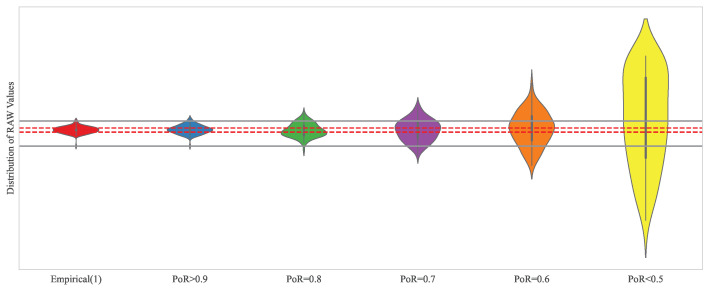
Distribution of the raw value of time-series data depending on PoR score: The grey line indicates the range of observed PoR values, highlighting maximum and minimum values, while the red dotted lines represent the 25th to 75th percentile range.

The proposed PoR metric enables quantitative comparisons between generated and empirical data by reflecting the time-series characteristics of the data with their descriptive statistics. To examine the composition of the data values for each PoR value, we generated synthetic data for specific sections of an arbitrary dataset and displayed the resulting PoR values in Figure 7.

Figure 7 shows that the values most similar to the empirical data appear when the PoR exceeds 0.9. As the PoR decreases from 0.9 to 0.5 and lower, the synthetic data increasingly fails to reflect the empirical data characteristics. If the PoR value decreases to 0.5 and lower, this indicates that the synthetic data resembles a random walk and no longer reflects the characteristics of the time series.

**Figure 8:**
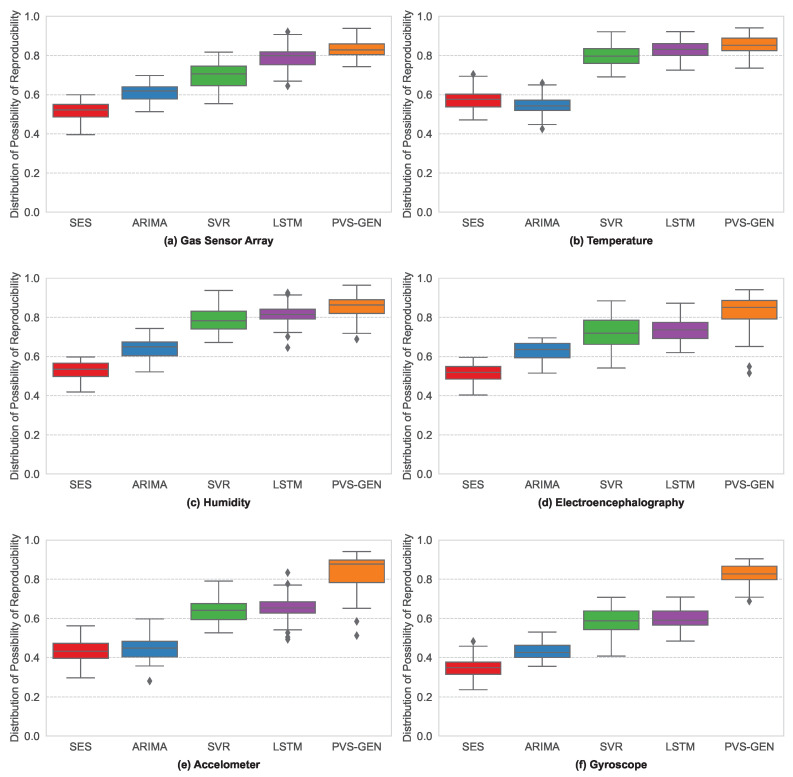
Distribution of PoR values for synthetic data generation across various sampled data types: (**a**–**c**) show Type-I time-series data, and (**d**–**f**) show Type-II time-series data.

We discussed how PoR values reflect the extent to which they represent the characteristics of the empirical data. We applied several synthetic data-generation methodologies to six randomly sampled data points from the four datasets previously introduced. Figure 8 shows the PoR values for both the generated synthetic and original empirical data. We used these values to assess how well the synthetic data-generation methodologies reflect the properties of the empirical data. By comparing the PoR values of the generated data with those of the original data, we can evaluate the effectiveness and quality of the synthetic data-generation methods. We comprehensively analyzed the results for different datasets and various synthetic data-generation techniques, including the SES, ARIMA, SVR, LSTM, and proposed PVS-GEN method.

Figure 8 shows the PoR values of synthetic data generated using various methods for different datasets. The x-axis indicates the methodology, and the y-axis indicates the PoR value. Figure 8a shows the results for gas sensor data; (b) and (c) for temperature and humidity, respectively; (d) for EEG data; and (e) and (f) for accelerometer and gyroscope data, respectively. As depicted in Figure 8, the PVS-GEN exhibits a high PoR distribution for all datasets. The average PoR distribution values are 0.53 (SES), 0.58 (ARIMA), 0.70 (SVR), 0.74 (LSTM), and 0.83 (PVS-GEN). Compared with other methods, PVS-GEN shows an increase in the PoR value of up to 37.1% and 19.1% on average. This indicates that the proposed method can generate data with higher reproducibility across various types of data. Notably, PVS-GEN exhibits a higher performance for more complex data types, such as Type-II, for which existing methodologies struggle to generate adequate data.

**Figure 9:**
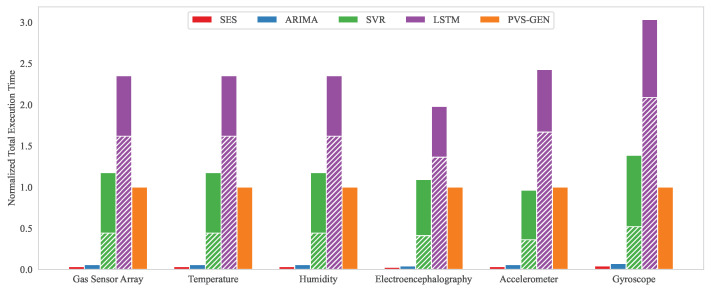
Total execution times normalized by PVS-GEN. Gas Sensor Array, Temperature and Humidity show Type-I time-series Data, while Electroencephalography, Accelerometer, and Gyroscope present Type-II time-series data. The hatch pattern at the bottom indicates training time, and the solid fill on top represents computational time.

The comparison of the time complexity between our proposed method and other algorithms is presented in Figure 9. To evaluate this, we conducted 5000 experiments, each measuring the execution time by PVS-GEN of each algorithm implemented on the same system, using randomly sampled subsets from the datasets. Figure 9 shows the normalized execution time by PVS-GEN of each methodology based on a random sample from seven datasets.

PVS-GEN demonstrates high reproducibility with a reasonable computation time. Additionally, it can independently reproduce the scales and characteristics of the data for segmented random intervals without being influenced by previous results. Although the SES and ARIMA methods maintain fixed computational loads irrespective of the model complexity when automatically extracting parameters, this results in a low reproducibility, as show in Figure 6 and Figure 8. Machine learning methodologies (SVR, and LSTM) require the most computational resources, resulting in longer execution times. Furthermore, when reproducing specific sampled data for training parameters or intervals, these methods exhibit scale distortion problems owing to the influence of previous results. In summary, the proposed PVS-GEN method demonstrates high reproducibility for specific extracted intervals, while maintaining lower time complexity, compared to machine learning techniques, irrespective of previous training or computational sequences.

A significant factor influencing the generation time and PoR value of data generated by PVS-GEN is the length of the data segments created during the segmentation process. More segmentations lead to more sections for data synthesis, which increases the overall execution time but may enhance the reproducibility of the synthetic data. Conversely, fewer segmentations result in a shorter overall execution time but are expected to decrease the reproducibility of the generated data.

**Figure 10:**
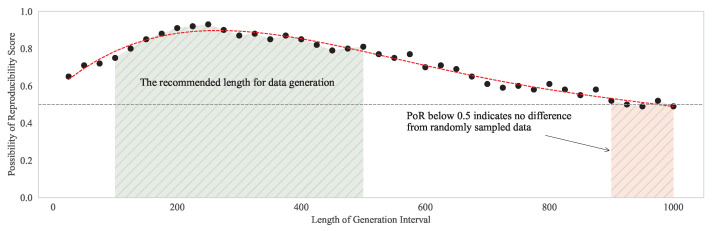
Average PoR values according to the length of the generated synthetic data (10,000 runs).

Figure 10 illustrates the average trend of the PoR values concerning the length of the parameterization and data-generation processes. The gray dashed line at y = 0.5 indicates a cutoff value; the data below this are considered meaningless. The generation intervals were from a minimum of 25 to a maximum of 1000, in increments of length 2, and the resulting trendline shows that as the data length increases, the PoR value initially increases but then decreases. This indicates that the optimal length for a single segmentation is approximately 250. Even when adjusting the segment length, applying a threshold or conducting preliminary segmentations on a single segment can be beneficial, with lengths ranging from a minimum number of points to a specified maximum. This approach helps avoid the pitfalls of sections that are either too short or too long, ensuring a balance between the execution time and data reproducibility.

## 5. Conclusions

Synthetic data generation has gained popularity as it reduces the cost and time required for acquiring and labeling large amounts of empirical data, allowing for diverse, tailored datasets that enhance model performances and address privacy concerns. However, time-series data generation faces numerous challenges owing to complex and diverse data characteristics. To address these challenges, the PVS-GEN process parameterizes empirical time-series data, verifies each generated synthetic data point, and segments intervals if the previous process cannot obtain valid parameters. Additionally, we can quantify the quality of the generated data by evaluating their time-series characteristics using the proposed PoR metric. In our experiments, the PVS-GEN process yielded a mean PoR value of 0.83, indicating an enhancement in reproducibility of up to 37.1% and an average increase of 19.1% compared with alternative methods, regardless of the type of empirical data. These results demonstrate that the PVS-GEN process can generate universal data with high reproducibility regardless of the sensor type.

In future work, we will continue to explore the modeling approach to further optimize the performance and improve the quality of the proposed generation methodologies. Furthermore, we plan to concentrate on optimizing the detailed components of various techniques, such as in data generation, parameter extraction, and change-point detection, to minimize the time overhead and ensure a high reproducibility.

## Figures and Tables

**Table 1:** Comparison of Type-I and Type-II time-series data types.

	Frequency	Variance *	Regularity
Type-I time series (low volatility)	Low	Low	High
Type-II time series (high volatility)	High	High	Low

* Variance refers to the degree of change in data values over a specific period.

**Table 2:** Comparative review of synthetic data generations.

Category	Proposal	Base Methodology	Target Data	Features
Statistical Based Model	ARIMA Model for predict Flood Risk [28]	Full-Series ARIMA, BMA method	Yalong River Basin Flood	Utilizes ARIMA for synthesizing future flood series, integrating past and predicted data.
	Duplex Markov Models [36]	Markov Chain	Wind Speed	Applies three Markov models for accurate high-resolution wind speed data generation.
	Modified VICAR [37]	VICAR	Gearbox Vibration Signals	Produces synthetic signals for improved detection of faults in gearboxes using VICAR
Machine Learning Based Model	Deep Learning Model for Solar Energy Prediction [39]	CNN-LSTM	Solar Irradiance, PV Power	Creates predictive synthetic data for solar energy using CNN-LSTM Model
	LSTM Model for COVID-19 Forecasting [45]	LSTM	COVID-19 Case Data in Canada	Generates forecasted synthetic data for spread of COVID-19 and their intervention impact
	GAN model for Electricity Consumption [47]	RCGAN, TimeGAN, CWGAN, RCWGAN	Electricity Consumption	Synthesizes electricity consumption data for smart grids using various GANs.

**Table 3:** Dataset specifications for experiments.

Dataset	Sensor Type	Data Range	Unit	Dataset Size
Gas Sensor Array	Gas Sensor Array	0∼$2^{7}$	ppm	284.58 MB
Low-Energy House	Temperature	$-2^{7}$∼$2^{7}$	°C	11.42 MB
	Humidity	0∼$2^{7}$	%R.H.	
EEG Alcoholism	Electroencephalography	$-2^{8}$∼$2^{8}$	mV	762.2 MB
Heterogeneity Activity Recognition	Accelerometer	$-2^{4}$∼$2^{4}$	g	3.07 GB
	Gyroscope	$-2^{4}$∼$2^{4}$	rad/s	

## Data Availability

Data are contained within the article.
